# Eating Disorders among Adolescent Female Students in Jeddah, Saudi Arabia

**DOI:** 10.21315/mjms2023.30.1.16

**Published:** 2023-02-28

**Authors:** Reem Almutairi, Ahmad Ariffin Azuhairi, Aidalina Mahmud, Anas S Dablool

**Affiliations:** 1 Department of Community Health, Faculty of Medicine and Health Sciences, Universiti Putra Malaysia, Selangor, Malaysia; 2 Department of Public Health, Health Sciences College at Al-Leith, Umm Al-Qura University, Makkah, Saudi Arabia

**Keywords:** eating disorder, school girls, eating attitude test-26

## Abstract

**Background:**

Eating disorders (EDs) are characterised by an abnormal attitude towards food that causes someone to change their eating habits and behaviour. This study aimed to investigate the prevalence of EDs and their associated factors among female going-school adolescents in Jeddah, Saudi Arabia.

**Methods:**

A cross-sectional study was conducted in five schools in Jeddah, Saudi Arabia, among a representative random sample of female adolescent students aged 13 years old–18 years old. A simple random sampling method was used to select the participants. An online self-administered questionnaire, the Arabic version of eating attitude test (EAT-26) and socio-cultural attitudes toward appearance questionnaire (SATAQ-4), was used.

**Results:**

More than half (53.6%) of adolescent girls scored at or above the cut-off point of EAT-26. Around 45% of the participants had experienced family influence on their appearance and body shape, 36.7% had experienced peer influence on these factors, and 49.4% had experienced media influence. Family influence was significantly associated with EDs (*P* = 0.013).

**Conclusion:**

The high prevalence of EDs among female going-school adolescents in Jeddah, Saudi Arabia, is of great concern. To mitigate this problem, effective programmes must be designed to change their dietary habits while considering the effects of family, peer and media influence, as well as focusing on the importance of eating breakfast and practising physical activity.

## Introduction

Eating disorders (EDs) are psychological and nutritional disorders that include the disturbance of eating behaviours and attitudes (1). Typically, people with EDs are primarily concerned with food and body image, and such affected people consider themselves obese, even if they are thin. The psychological and nutritional consequences of EDs include malnutrition, osteoporosis, menopause, cardiovascular disease and depression, while the severe clinical consequences include anorexia nervosa and bulimia nervosa (2). Adolescent girls are at great risk of suffering from EDs. Such individuals are prone to numerous health problems attributed to their lifestyles and eating habits (3, 4). EDs occur mainly in Western countries, especially among young women aged 15 years old–24 years old; however, over recent years, EDs have also been increasing in Eastern cultures; they occur across all ethnic, cultural and socioeconomic groups (5–7). The prevalence of EDs in Western countries was approximately 0.3%–1% among young adolescent girls. In Eastern countries, the prevalence of EDs varied from 6% to 49.1% among adolescents from Arab countries, and the rate was higher among females than males. The highest prevalence was reported in Emirati and Saudi adolescents (6, 8). EDs are believed to arise from the interaction of multiple risk factors. Previous studies have found that socio-cultural factors are associated with ED behaviour among adolescents (9, 10). Family, peers, and the media are the most critical socio-cultural factors that could form potential risk factors for EDs. Adolescents are exposed to social pressures from their family, friends and the media concerning their appearance and body shape, as well as the need to lose weight, which predisposes this population to develop dissatisfaction with their body image, leading to EDs; this reflects the vulnerability of this population (11). A study found that social appearance anxiety partly mediated the association between low self-confidence, ED risk and body esteem among female adolescents (12). The prevalence of EDs among females increased from 3.5% in 2000–2006 to 7.8% in 2013–2018, making this condition an alarming issue for public health services and healthcare providers (13). However, there remains a lack of studies investigating EDs in relation to their prevalence and association with socio-cultural factors in Saudi Arabia. Therefore, this study aims to estimate the prevalence of EDs and the associated factors among female going-school adolescents in Jeddah, Saudi Arabia. The findings from this study will develop the overall understanding of the prevalence of EDs in Saudi Arabia, contributing to the advancement of knowledge on this issue and benefitting future studies.

## Methods

### Study Design, Sampling and Participants

A cross-sectional study was conducted in five public schools in Jeddah, Saudi Arabia, among female adolescent students (aged 13 years old–18 years old) in that city who agreed to participate in the study. Students with physical or psychological disabilities were excluded from this study. The sampling frame was a list of student names of selected schools in Jeddah, Saudi Arabia for the period from June 2021 until July 2021. The sample size was calculated using a single proportion sampling formula, based on the previously identified prevalence of EDs among adolescent girls in Arar City, Saudi Arabia (14). The total sample size was 554 subjects after considering a power of 80%, a 95% confidence level, and a 30% estimate of incomplete data.

### Sampling Technique

Five schools were selected using the stratified random sampling technique by dividing Jeddah into five areas to ensure that the study was representative of the whole population. In total, 344 Jeddah schools were identified in this way. After numbering pieces of paper symbolising each area’s schools, one paper was randomly selected. After identifying the schools from each area, the school administration was approached personally with an approval letter from the Ministry of Education in Saudi Arabia. The researcher explained the purpose of the study to the school administrators. Based on the approval and cooperation of the school administrators, one school was randomly selected from each area. The sampling unit was each student who met the inclusion criteria between June and July 2021. The sample sizes were apportioned based on the size of the school. Student name lists for all the classes were obtained from the school administration at each school. Then, the names of the students in the sampling frame were numbered. A proportional stratified sampling technique (PSS) was used to select the participants according to the density of the students in each school. A total of 554 students were selected according to the inclusion and exclusion criteria; 155 participants were selected from the 100 schools, 108 participants from the 55 schools, 90 participants from the 40 schools, 56 participants from the 84 schools and 145 participants from the 92 schools.

### Data Collection

After obtaining the necessary permission to conduct the study, each school principal was given consent forms to distribute via the students’ and parents’ emails, so that the students and their parents could sign to give their consent. Signed consent forms were obtained from both the students and their parents before the subjects participated in the study. An online session was conducted during data collection to explain the purpose of the survey. A set of self-administered questionnaires was then distributed to the respondents from June 2021 until July 2021 via Google Forms. All the respondents were required to complete the self-administered questionnaire after the researcher had explained how to do this. The study instruments included four Arabic version questionnaires: a socio-demographic questionnaire (detailing age, nationality, parents’ education level, and parents’ monthly income), the eating attitude test (EAT-26) questionnaire and the socio-cultural attitudes toward appearance (SATA-4) questionnaire. The questionnaire was translated to Arabic, where two language experts were validated the translation of the questionnaire to the Arabic language.

The EAT-26 was used to collect data on the EDs prevalence, based on the work of Garner (15). The EAT is a 26-item questionnaire that is widely used to measure the symptoms and concerning characteristics of EDs in adults and adolescents aged 14 years old and above. There were three main subscales of the EAT-26: dieting (13 items), bulimia and food preoccupation (6 items) and oral control (7 items). The items are rated on a six-point Likert scale from ‘always’ (6) to ‘never’ (1). The items from 1 to 25 have six classification points from (always) to (never). A score of 3 was given for (always), a score of 2 was given for (usually), a score of 1 was given for (often) and a score of 0 was given for (sometimes), (rarely) and (never). Item 26 scored 0 for (always), (usually) and (often); it scored 1 for (sometimes), 2 for (rarely) and 3 for (never). A total score of 20 or above is considered to indicate an ED (3). The reliability of the EAT-26 was 0.87, based on Cronbach’s *α*. The SATA-4 was used to collect data on environmental factors (family influence, peer influence and media influence), as developed by Schaefer et al. (16). The family influence scale has four items that assess the attitudes of an individual’s family towards appearance. It also measures a family’s focus on appearance and body shape. The family includes the subject’s parents, brothers, sisters and other relatives. The peer influence scale has four items that assess the attitudes of a subject’s peers towards appearance or their peers’ focus on appearance and body shape. Peers include their close friends, classmates and other social contacts. The media influence scale has four items that assess the attitudes of the media towards appearance and body shape. The media includes television, magazines, the internet, movies, billboards and advertisements. The respondents were required to rate each item on a 5-point Likert scale with responses ranging from ‘strongly disagree’ (1) to ‘strongly agree’ (5). Reverse scoring was used for the present study so that higher scores represented more problematic family attitudes. Higher scores indicated higher levels of perceived pressure from parents, peers and the media to gain weight, lose weight or increase muscle tone (17).

### Data Analysis

Data was analysed using the software Statistical Package for Social Sciences (SPSS) version 26.0 for Microsoft Windows (Chicago, IL, USA). The data is presented by descriptive statistics to determine means, medians and standard deviations (SD) (for continuous variables), while frequencies and percentages represented categorical data (the categorical variables). The chi-squared test was used to determine the association between the variables of the study. The significance level for statistics was determined at *P* < 0.05. Univariate logistic regression analysis was conducted and a crude odds ratio (COR) was determined. Any variable having a significant univariate test was selected as a candidate for the multivariate analysis. The significance level for the statistics was deemed acceptable at 0.05.

This study was approved by the Ethics Committee for Research Involving Human Subjects (JKEUPM) of Universiti Puta Malaysia [UPM/TNCPI/RMC/JKEUPM/1.4.18.2 (JKEUPM)] and by the Ministry of Education in Saudi Arabia. Written consent was obtained from all the participants from all the schools and the students’ parents, and they received written information about the study before data collection began. The participants and their parents had the opportunity to ask questions about the study before giving their consent.

## Results

In the present study, 502 students were included for data analysis purposes. Table 1 summarises their socio-demographic characteristics. Most participants (64.5%) were within the age range of 16 years old–18 years old. The participants’ ages ranged from 13 years old–18 years old. The overall median age (IQR) was 16 (2). Most participants were Saudi (90.8%). Most of the participants’ fathers (44.2%) had undergraduate degrees or had reached higher education, while most mothers (38.4%) had had high school education. Among the participants, 47.8% had a moderate monthly income (5,000–14,999 Saudi Riyal). Most participants (65.1%) were in the secondary grade. Most were from the One-hundred School (26.3%) followed by the Ninety-two School (26.1%), the Fifty-five School (20.1%), the Forty School (17.7%) and the Eighty-four School (9.8%).

**Figure 1:**
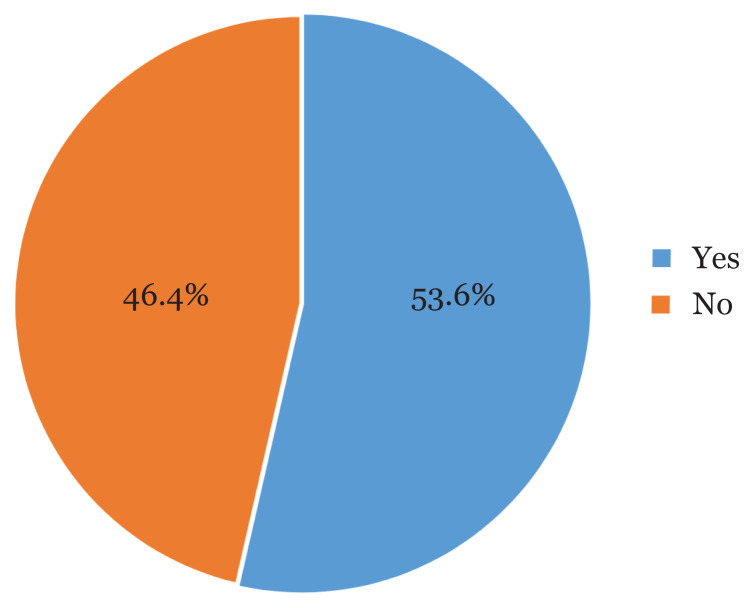
The prevalence of eating disorder among the participants

The EAT-26 questionnaire was used to measure the symptoms and concerning characteristics of EDs among the participants. The median (IQR) of the EAT-26 score was 21 (22). Most of the adolescent female students (269 or 53.6%) scored at or above the cut-off point of EAT-26 (20), indicating that the prevalence of EDs among the participants was 53.6% (Figure 1).

Table 2 presents the eating attitudes and EAT-26 characteristics of the participants. Among the participants, 24.9% were always terrified about being overweight, whereas 25.9% rarely avoided eating when they were hungry. Around 27% of them were sometimes preoccupied with food, while 23.9% often found themselves preoccupied with food. Approximately 58% had never gone or often went on eating binges when they felt that they might not be able to stop, whereas 21.3% said this always or usually applied to them. In all, 38.4% of the girls always or usually cut their food into small pieces, compared to 22.1% who said they never or rarely did. Most participants (47.7%) were never or rarely aware of the calorie content of the foods they ate, whereas 26.3% were always or usually aware of the calorie content. Around 44% of the girls never or often particularly avoided food with high carbohydrate content, compared to 25.7% who said they always or usually did. In all, 24.9% of the girls reported that they always felt that others would prefer it if they ate more and 17.5% reported never feeling that. More than half of them (51.2%) never vomited after eating, compared to 16.3% who always did. More than a quarter of the schoolgirls (30.3) never felt extremely guilty after eating. However, 21.3% always felt so. More than a quarter of the schoolgirls (35.3%) were always preoccupied with a desire to be thinner, whereas only 11.6% of them were never preoccupied with that. In addition, 34.9% of them always thought about burning up calories when they exercised, compared to 14.7% who never thought about that. Around 40% of the participants always or usually believed that other people thought they were too thin, whereas 34% rarely or never believed this. In all, 36.8% of them were always or usually preoccupied with the thought of having fat on their body, compared to 35.6% who were rarely or never preoccupied with that. Most of the schoolgirls (37.7%) always or usually took longer than others to eat their meals, whereas 29% of them rarely or never did. Overall, 40% of the girls rarely or never avoided foods containing sugar, compared to 26.5% who always or usually avoided such food. More than half of them (50.2%) rarely or never eat diet foods, compared to 20.7% who always or usually did. The majority of the girls (40.6%) rarely or never felt that food controlled their life, whereas 28.5% always or usually felt like that. Most of the schoolgirls (54.6%) always or usually displayed self-control around food, compared to only 16% who rarely or never did. Around 38% of them always or usually felt that others pressured them to eat, compared to 34.6% who rarely or never felt that. In all, 47.2% of the participants rarely or never gave too much time and thought to food, whereas 24.9% always or usually spent too much time thinking about food. Most of them (40.4%) rarely or never felt uncomfortable after eating sweets, whereas 29.5% always or usually felt that. In total, 34.1% of them never engaged in dieting behaviour, compared to the 18.9% who rarely did so and the 18.3% who always engaged in dieting behaviour. Overall, 19.7% of them reported that they sometimes liked their stomach to be empty, compared to 19.1% who said they always did and 13.5% who said they never liked that. Most of them (43.8%) never had the impulse to vomit after meals, whereas 17.3% of them always had this impulse. In all, 22.9% of the schoolgirls often enjoyed trying new rich foods, compared to 21.7%, 21.1% and 13.1% who always, sometimes and never enjoyed that, respectively.

Table 3 shows the characteristics of the socio-cultural attitudes identified from the survey. The majority of the participants definitely disagreed that they felt pressure from their family members to look thinner (54.6%); some (47.6%) felt pressure from family members to improve their appearance, and some (32.9%) were encouraged by their family members to reduce their level of body fat. Meanwhile, most participants definitely agreed (35.1%) that family members encouraged them to get into better shape. Most participants reported that they definitely disagreed with their peers when the latter encouraged them to become thinner; that they felt pressure from their peers to improve their appearance; that they felt pressure from their peers to look in better shape, and that they felt pressure from their peers to decrease their level of body fat, with scores of 43.6%, 62.0%, 63.7% and 66.7%, respectively. Most participants definitely disagreed that they felt pressure from the media to look in better shape; that they felt pressure from the media to look thinner; that they felt pressure from the media to improve their appearance; and that they felt pressure from the media to decrease their level of body fat, with scores of 58.2%, 61.4%, 56.6% and 59.4%, respectively.

Table 4 presents the association between socio-demographic characteristics and EDs. Of the participants in the 16 years old–18 years old of age group, 56.2% had EDs, compared to 48.9% in the 13 years old–15 years old of age group, and this association was not significant (*χ^2^* = 2.5, *P* = 0.117). Nationality (*χ^2^* = 2.1, *P* = 0.150), father’s education level (*χ^2^* = 1.2, *P* = 0.801), mother’s education level (*χ^2^* = 1.5, *P* = 0.395), monthly income (*χ^2^* = 3.4, *P* = 0.105), grade (*χ^2^* = 0.8, *P* = 0.370) and school name (*χ^2^* = 5.7, *P* = 0.682) were not significantly associated with EDs. Family influence was significantly associated with EDs (OR =1.072; 95% CI =1.015, 1.133; *P* = 0.013), but there was no significant association between peer influence (OR = 1.025; 95% CI =0.973, 1.080; *P* = 0.357) and media influence (OR =1.012; 95% CI = 0.981, 1.045, *P* = 0.437) with EDs (Table 5).

To detect the predictors of EDs among female adolescent students, logistic regression was applied. As a preliminary model, all the variables were identified using univariate logistic regression one by one independently. Five variables (age, nationality, school name, household monthly income and family influence) turned out to be significant. The factor that predicted ED among female adolescent’s students was family influence (OR = 0.067; 95% CI = 1.010, 1.131; *P* = 0.021) (Table 6).

## Discussion

This study showed that approximately half of the participants (53.6%) scored at or above the cut-off point of the EAT-26 (20), indicating the prevalence of EDs among the participants was 53.6%. The prevalence of disordered eating was higher than has previously been reported among adolescent schoolgirls in Saudi Arabia and Arab countries (18–21). In general, adolescent females in Saudi Arabia are subjected to abrupt lifestyle pattern shifts due to social and developmental changes that include a shift towards urban living. These changes will drive teenage females to acquire certain negative attitudes as they try to keep pace with the modernising process, such as unhealthy approaches to eating and maintaining their body weight (22). Furthermore, the difference between the current study findings and the results from studies undertaken in other Arab countries may be attributed to the difference in the study duration, since this study was conducted during the COVID-19 pandemic. The COVID-19 pandemic and the lockdown has forced an abrupt change in individual eating and exercise behaviours, including among female adolescents. Emerging evidence suggests that the COVID-19 pandemic may negatively impact eating and exercise behaviours (23). This may be because adolescents are confined to their homes, causing decreased physical activity, far more extended periods of television time, irregular sleep patterns, less favourable diets and weight gain (24).

Although more participants in the 16 years old–18 years old of age group had EDs (56.2%) than those in the other group, age was not significantly associated with EDs among female adolescents in this study. Similar findings from other studies in Saudi Arabia among adolescents identified no significant association between EDs and age group (14, 22). In addition, 54.6% of the Saudi girls had EDs, compared to 43.5% of the non-Saudis; however, this association was not significant (*P* = 0.1). These findings were consistent with other studies that reported no significant association between EDs and nationality (7, 22). However, a study in Oman found that Omani adolescents were more significantly associated with EDs than non-Omani adolescents (19). Differences in cultures and eating habits might explain these variations since each culture and country has different meal habits and dietary intake patterns. The findings from this study show no significant association between EDs and parental education level (*P* > 0.05). This result aligns with a study conducted by Alwosaifer et al. (25) in Saudi Arabia, which found no significant association between EDs and parental education (*P* = 0.10). However, these findings are contrary to a study by Sundquist et al. (26), which found a significant association between high parental education and increased risk of EDS among female adolescents. Additionally, another study found that higher parental and grandparental education levels were significantly associated with a raised risk of EDs among female adolescents (27). Furthermore, no significant association between EDS and household monthly income was identified in this study. Similarly, a study among adolescents in Saudi Arabia reported no significant association between EDs and household monthly income (25). This contrasts with a study by Hunger and Tomiyama (28), which reported a significant association between EDs and household monthly income (*P* < 0.05). The influences of social and family circumstances, such as household monthly income, play a significant role in developing children’s behaviours and increased parental care might explain these findings.

Adolescents are exposed to social pressures from family, friends and the media that predispose this population to develop dissatisfaction with their body image, leading to EDs; this reflects the vulnerability of this population (29). In this study, 45.6% of the adolescent students showed a high median score of family influence, while family influence was a significant predictor of the risk of developing an ED among the students in this study. This result is consistent with a study conducted in Saudi Arabia showing that around 50% of female students had been influenced by their family to lose weight (25). Although some female adolescents had higher median scores for peer and media influences on their appearance, body shape and weight loss, (36.7% and 49.4%, respectively), neither peer nor media influence had a significant association with EDs. Similar results have been reported by studies that have linked parental influence with increased incidence of disordered eating (17, 30, 31). Parents’ comments and criticisms relating to body shape and weight have been linked to body image concerns and the development of EDs among female adolescents (32, 33). In contrast, another study reported a significant association between EDs and responsiveness to mass media; that is, exposure to television and magazine images of thin celebrities and models was found to play a role in the development of body shape concerns and dissatisfaction among female adolescents, which in turn increases their risk of developing EDs (17). Research also shows that adolescent females might be more sensitive to, and influenced by, their peers’ comments about their appearance and weight than those in other cultural environments (34).

There are some limitations of this study. The main limitation is that BMI calculations, including weight and height measurements, were not reported in this study because it was undertaken online via Google Forms because of the COVID-19 pandemic. Also, self-reported measures of weight and height may have caused inaccuracy owing to response bias. A second limitation was that the cross-sectional design showed the associations between risk factors but not the causation, so the results cannot be generalised. Collecting data using self-reported questionnaires is susceptible to bias.

## Conclusion

The high prevalence of disordered eating attitudes among schoolgirls in Jeddah, Saudi Arabia, is of great concern. In addition, the results indicated that family influence on female adolescents’ appearance and body shape, as well as its association with EDs, provide grounds for concern. To mitigate these problems, effective programs must be designed to change their dietary habits while considering the effects of family, peer and media influence, as well as focusing on the importance of eating breakfast and practising physical activity.

## Figures and Tables

**Table 1:** Distribution of socio-demographic and economic characteristics of the participants (*N* = 502)

Variables	Frequency	Percentage (%)
Age (years old), Media (IQR)	16 (2)	
13–15	178	35.5
16–18	324	64.5
Nationality		
Saudi	456	90.8
Non-Saudi	46	9.2
Father education level		
Intermediate school or lower	101	20.1
High school	179	35.7
Undergraduate degree or higher	222	44.2
Mother education level		
Intermediate school or lower	125	24.9
High school	193	38.4
Undergraduate degree or higher	184	36.7
Monthly income (SR), Median (IQR)	9800 (9625)	
4,999	118	23.5
5,000–14,999	240	47.8
15,000	144	28.7
Grade		
Intermediate	175	34.9
Secondary	327	65.1
School Name		
The One-hundred School	132	26.3
The Fifty-five School	101	20.1
The Forty School	89	17.7
The Eighty-four School	49	9.8
The Ninety-two School	131	26.1

Notes: IQR = interquartile range; SR = Saudi Riyal

**Table 2:** Distribution of eating attitudes characteristics of the participants (*N* = 502)

No.	Items	Always	Usually	Often	Sometimes	Rarely	Never
1.	Am terrified about being overweight.	125 (24.9)	65 (12.9)	75 (14.9)	84 (16.7)	93 (18.5)	60 (12.0)
2.	Avoid eating when I am hungry.	63 (12.5)	59 (11.8)	95 (18.9)	93 (18.5)	130 (25.9)	62 (12.4)
3.	Find myself preoccupied with food.	54 (10.8)	50 (10)	120 (23.9)	135 (26.9)	78 (15.5)	65 (12.9)
4.	Have gone on eating binges where I feel that I may not be able to stop.	71 (14.1)	36 (7.2)	41 (8.2)	62 (12.4)	140 (27.9)	152 (30.3)
5.	Cut my food into small pieces.	108 (21.5)	85 (16.9)	115 (22.9)	83 (16.5)	74 (14.7)	37 (7.4)
6.	Aware of the calorie content of foods that I eat.	91 (18.1)	41 (8.2)	64 (13.3)	67 (13.3)	125 (24.9)	114 (22.7)
7.	Particularly avoid food with a high carbohydrate content (i.e. bread, rice, potatoes, etc.)	76 (15.1)	53 (10.6)	77 (15.3)	74 (14.7)	130 (25.9)	92 (18.3)
8.	Feel that others would prefer if I ate more.	125 (24.9)	50 (10.0)	76 (15.1)	72 (14.3)	91 (18.1)	88 (17.5)
9.	Vomit after I have eaten.	82 (16.3)	24 (4.8)	29 (5.8)	38 (7.6)	72 (14.3)	257 (51.2)
10.	Feel extremely guilty after eating.	107 (21.3)	36 (7.2)	50 (10)	66 (13.1)	91 (18.1)	152 (30.3)
11.	Am preoccupied with a desire to be thinner.	177 (35.3)	62 (12.4)	63 (12.5)	52 (10.4)	90 (17.9)	58 (11.6)
12	Think about burning up calories when I exercise.	175 (34.9)	65 (12.9)	61 (12.2)	64 (12.7)	63 (12.5)	74 (14.7)
13.	Other people think that I am too thin.	138 (27.5)	65 (12.9)	87 (17.3)	88 (17.5)	41 (8.2)	83 (16.5)
14.	Am preoccupied with the thought of having fat on my body.	119 (23.7)	66 (13.1)	61 (12.2)	77 (15.3)	107 (21.3)	72 (14.3)
15.	Take longer than others to eat my meals.	127 (25.3)	62 (12.4)	76 (15.1)	91 (18.1)	80 (15.9)	66 (13.1)
16.	Avoid foods with sugar in them.	84 (16.7)	49 (9.8)	74 (14.7)	94 (18.7)	103 (20.5)	98 (19.5)
17.	Eat diet foods.	76 (15.1)	28 (5.6)	71 (14.1)	75 (14.9)	125 (24.9)	127 (25.3)
18.	Feel that food controls my life.	102 (20.3)	41 (8.2)	79 (15.7)	76 (15.1)	109 (21.7)	95 (18.9)
19.	Display self-control around food.	176 (35.1)	98 (19.5)	90 (17.9)	58 (11.6)	43 (8.6)	37 (7.4)
20.	Feel that others pressure me to eat.	135 (26.9)	56 (11.2)	69 (13.7)	68 (13.5)	77 (15.3)	97 (19.3)
21.	Give too much time and thought to food.	73 (14.5)	52 (10.4)	57 (11.4)	83 (16.5)	117 (23.3)	120 (23.9)
22.	Feel uncomfortable after eating sweets.	109 (21.7)	39 (7.8)	78 (15.5)	73 (14.5)	86 (17.1)	117 (23.3)
23.	Engage in dieting behaviour.	92 (18.3)	41 (8.2)	56 (11.2)	47 (9.4)	95 (18.9)	171 (34.1)
24.	Like my stomach to be empty.	96 (19.1)	63 (12.5)	86 (17.1)	99 (19.7)	90 (17.9)	68 (13.5)
25.	Have the impulse to vomit after meals.	87 (17.3)	35 (7.0)	36 (7.2)	35 (7.0)	89 (17.7)	220 (43.8)
26.	Enjoy trying new rich foods.	109 (21.7)	70 (13.9)	115 (22.9)	106 (21.1)	36 (7.2)	66 (13.1)

**Table 3:** Distribution of sociocultural attitudes characteristics (*N* = 502)

No.	Items	Scale *n* (%)				

		5 = definitely agree	4	3	2	1 = definitely disagree
**Family influences**						
Median (IQR) = 13 (4)						
1.	I feel pressure from family members to look thinner.	75 (14.9)	48 (9.6)	49 (9.8)	56 (11.2)	274 (54.6)
2.	I feel pressure from family members to improve my appearance.	82 (16.3)	62 (12.4)	56 (11.2)	63 (12.5)	239 (47.6)
3.	Family members encourage me to decrease my leve of body fat.	116 (23.1)	74 (14.7)	95 (18.9)	52 (10.4)	165 (32.9)
4.	Family members encourage me to get in better shape.	176 (35.1)	81 (16.1)	74 (14.7)	54 (10.8)	117 (23.3)

**Peer influences**						
Median (IQR) = 16 (4)						
5.	My peers encourage me to get thinner.	93 (18.5)	66 (13.1)	80 (15.9)	44 (8.8)	219 (43.6)
6.	I feel pressure from my peers to improve my appearance.	51 (10.2)	24 (4.8)	62 (12.4)	54 (10.8)	311 (62.0)
7.	I feel pressure from my peers to look in better shape.	52 (10.4)	27 (5.4)	52 (10.4)	51 (10.2)	320 (63.7)
8.	I get pressure from my peers to decrease my level of body fat.	54 (10.8)	16 (3.2)	51 (10.2)	46 (9.2)	335 (66.7)

**Media influences**						
Median (IQR) = 20 (8)						
9.	I feel pressure from the media to look in better shape.	69 (13.7)	48 (9.6)	46 (9.2)	47 (9.4)	292 (58.2)
10.	I feel pressure from the media to look thinner.	62 (12.4)	46 (9.2)	47 (9.4)	39 (7.8)	308 (61.4)
11.	I feel pressure from the media to improve my appearance.	69 (13.7)	47 (9.4)	58 (11.6)	44 (8.8)	284 (56.6)
12.	I feel pressure from the media to decrease my level of body fat.	62 (12.4)	44 (8.8)	56 (11.2)	42 (8.4)	298 (59.4)

**Table 4:** Association between sociodemographic characteristics and eating disorders

Variables	Eating disorder		*χ* *^2^*	*P*-value

	Yes	No		

	*n* (%)	*n* (%)		
Age (years old)				
13–15	87 (48.9)	91 (51.1)	2.5	0.117
16–18	182 (56.2)	142 (43.8)		
Nationality				
Saudi	249 (54.6)	207 (45.4)	2.1	0.150
Non-Saudi	20 (43.5)	26 (56.5)		
Father education level				
Intermediate school or lower	56 (55.4)	45 (44.6)	1.2	0.801
High school	90 (50.3)	89 (49.7)		
Undergraduate degree or higher	23 (55.4)	99 (44.6)		
Mother education level				
Intermediate school or lower	66 (52.8)	59 (47.2)	1.5	0.395
High school	98 (50.8)	95 (49.2)		
Undergraduate degree or higher	105 (57.1)	79 (42.9)		
Monthly income				
4,999	60 (50.8)	58 (49.2)	3.4	0.105
5,000–14,999	122 (50.8)	118 (49.2)		
15,000	87 (6.4)	57 (39.6)		
Grade				
Intermediate	89 (50.9)	86 (49.1)	0.8	0.370
Secondary	180 (55.0)	147 (45.0)		
School name				
The one-hundred school	72 (54.5)	60 (45.5)	5.7	0.682
The fifty-five school	49 (48.5)	52 (51.5)		
The forty school	57 (64.0)	32 (36.0)		
The eighty-four school	25 (51.0)	24 (49.0)		
The ninety-two school	66 (50.4)	65 (49.6)		

Notes:

* significance *P* < 0.05;

χ^2^ = chi-square statistic

**Table 5:** Association between sociocultural attitudes and eating disorders

Variables	Eating Disorder		OR^a^	95% CI^b^	*P*-value

	Yes Median (IQR)	No Median (IQR)			
Family influences scores	13 (4)	14 (4)	1.072	1.015–1.133	0.013*
Peer influences scores	16 (4)	16 (3)	1.025	0.973–1.080	0.357
Media influences scores	18 (8)	20 (8)	1.012	0.981–1.045	0.437

Notes:

a OR = crude odds ratio;

b 95% CI = confidence interval;

* significance *P* < 0.05

**Table 6:** The predictors of eating disorder among female adolescent students

Variables	B^a^	SE^b^	Wald	df^c^	*P*-value	AOR^d^	95% CI for OR^e^	

							Lower	Upper
Age group (years old)								
[13–15]	1							
16–18	−0.472	0.384	1.504	1	0.220	0.624	0.294	1.326
Nationality								
Saudi	−0.354	0.319	1.232	1	0.267	0.702	0.375	1.311
[Non-Saudi]	1							
School name								
The one-hundred school	0.220	0.424	0.269	1	0.604	1.246	0.542	2.863
The fifty-five school	0.325	0.410	0.628	1	0.428	1.383	0.620	3.087
The forty school	−0.131	0.453	0.083	1	0.773	0.878	0.361	2.131
The eighty-four school	0.026	0.341 0.006	1	0.940	1.026	0.526	2.004	
[The ninety-two school]	1							
Household monthly income								
4,999	0.389	0.265	2.153	1	0.142	1.476	0.878	2.482
5,000–14,999	0.337	.221	2.329	1	0.127	1.401	0.909	2.160
15,000]	1							
Family influence	0.067	0.029	5.327	1	0.021	1.069	1.010	1.131

Notes: [ ] = reference group;

a B = coefficient for adjusted OR;

b SE = standard error;

c df = degree of freedom;

d AOR = adjusted odds ratio;

e 95% CI = confidence interval;

* *P*-value = significant at *P* < 0.05

## References

[1] Hay P, Mitchison D, Collado AEL, González-Chica DA, Stocks N, Touyz S (2017). Burden and health-related quality of life of eating disorders, including Avoidant/Restrictive Food Intake Disorder (ARFID), in the Australian population. J Eat Disord.

[2] Plichta M, Jezewska-Zychowicz M (2020). Orthorexic tendency and eating disorders symptoms in Polish students: examining differences in eating behaviors. Nutrients.

[3] Musaiger AO, Al-Mannai M, Tayyem R, Al-Lalla O, Ali EYA, Kalam F (2013). Risk of disordered eating attitudes among adolescents in seven Arab countries by gender and obesity: a cross-cultural study. Appetite.

[4] Galmiche M, Déchelotte P, Lambert G, Tavolacci MP (2019). Prevalence of eating disorders over the 2000–2018 period: a systematic literature review. Am J Clin Nutr.

[5] Chang YJ, Lin W, Wong Y (2011). Survey on eating disorder–related thoughts, behaviors, and their relationship with food intake and nutritional status in female high school students in Taiwan. J Am Coll Nutr.

[6] Melisse B, de Beurs E, van Furth EF (2020). Eating disorders in the Arab world: a literature review. J Eat Disord.

[7] Waseem F, Ahmad LM (2018). Prevalence of disordered eating attitudes among adolescent girls in Arar City, Kingdom of Saudi Arabia. Health Psychol Res.

[8] Brown Z, Tiggemann M (2021). Celebrity influence on body image and eating disorders: a review. J Health Psychol.

[9] Gan WY, Mohamad N, Law LS (2018). Factors associated with binge eating behavior among Malaysian adolescents. Nutrients.

[10] Eshak ES, Ghazawy ER, Mohammed ES (2020). Sociocultural attitudes toward appearance and body shape dissatisfaction in adolescent Egyptian females: association and moderators. Health Promot Int.

[11] Izydorczyk B, Sitnik-Warchulska K (2018). Sociocultural appearance standards and risk factors for eating disorders in adolescents and women of various ages. Front Psychol.

[12] Halliwell E, Harvey M (2006). Examination of a sociocultural model of disordered eating among male and female adolescents. Br J Health Psychol.

[13] Duncan AE, Ziobrowski HN, Nicol G (2017). The prevalence of past 12-month and lifetime DSM-IV eating disorders by BMI category in US men and women. Eur Eat Disord Rev.

[14] Fatima W, Ahmad LM (2018). Prevalence of disordered eating attitudes among adolescent girls in Arar City, Kingdom of Saudi Arabia. Health Psychol Res.

[15] Garner DM (2009). Screening and case finding for the general practitioner. EAT-26 self-test.

[16] Schaefer LM, Burke NL, Thompson JK, Dedrick RF, Heinberg LJ, Calogero RM (2015). Development and validation of the sociocultural attitudes towards appearance questionnaire-4 (SATAQ-4). Psychol Assess.

[17] Young EA, McFatter R, Clopton JR (2001). Family functioning, peer influence, and media influence as predictors of bulimic behavior. Eat Behav.

[18] Madanat HN, Lindsay R, Campbell T (2011). Young urban women and the nutrition transition in Jordan. Public Health Nutr.

[19] Al-Adawi S, Dorvlo ASS, Burke DT, Moosa S, Al-Bahlani S (2002). A survey of anorexia nervosa using the Arabic version of the EAT-26 and ‘gold standard’ interviews among Omani adolescents. Eat Weight Disord.

[20] Sepulveda AR, Todd G, Whitaker W, Grover M, Stahl D, Treasure J (2010). Expressed emotion in relatives of patients with eating disorders following skills training program. Int J Eat Disord.

[21] Fallatah A, Al-Hemairy M, Al-Ghamidi H (2015). Eating disorders among female adolescents in Jeddah. Scientific Cooperation.

[22] Allihaibi MM (2015). Disordered eating attitudes among secondary schoolgirls in Al-Iskan sector, Makkah Al-Mukarramah, Saudi Arabia. Int J Med Sci Public Health.

[23] Phillipou A, Meyer D, Neill E, Tan EJ, Toh WL, Van Rheenen TE (2020). Eating and exercise behaviors in eating disorders and the general population during the COVID-19 pandemic in Australia: initial results from the COLLATE project. Int J Eat Disord.

[24] Graell M, Morón-Nozaleda MG, Camarneiro R (2020). Children and adolescents with eating disorders during COVID-19 confinement: difficulties and future challenges. Eur Eat Disord Rev.

[25] Alwosaifer AM, Alawadh SA, Wahab MMA, Boubshait LA, Almutairi BA (2018). Eating disorders and associated risk factors among Imam Abdulrahman bin Faisal university preparatory year female students in Kingdom of Saudi Arabia. Saudi Med J.

[26] Sundquist J, Ohlsson H, Winkleby MA, Sundquist K, Crump C (2016). School achievement and risk of eating disorders in a Swedish national cohort. J Am Acad Child Adolesc Psychiatry.

[27] Litmanen J, Fröjd S, Marttunen M, Isomaa R, Kaltiala-Heino R (2017). Are eating disorders and their symptoms increasing in prevalence among adolescent population?. Nord J Psychiatry.

[28] Hunger JM, Tomiyama AJ (2018). Weight labeling and disordered eating among adolescent girls: longitudinal evidence from the National Heart, Lung, and Blood Institute growth and health study. J Adolesc Health.

[29] Ferrari EP, Petroski EL, Silva DAS (2013). Prevalence of body image dissatisfaction and associated factors among physical education students. Trends Psychiatry Psychother.

[30] Kluck AS (2010). Family influence on disordered eating: The role of body image dissatisfaction. Body Image.

[31] Hasan HA, Najm L, Zaurub S, Jami F, Javadi F, Deeb LA (2018). Eating disorders and body image concerns as influenced by family and media among university students in Sharjah, UAE. Asia Pac J Clin Nutr.

[32] Michael JE, Bulik CM, Hart SJ, Doyle L, Austin J (2020). Perceptions of genetic risk, testing, and counseling among individuals with eating disorders. Int J Eat Disord.

[33] Abraczinskas M, Fisak B, Barnes RD (2012). The relation between parental influence, body image, and eating behaviors in a nonclinical female sample. Body Image.

[34] Garrusi B, Baneshi MR (2013). Eating disorders and their associated risk factors among Iranian population—a community based study. Glob J Health Sci.

